# Complications associated with submental liposuction: a scoping review

**DOI:** 10.4317/medoral.25122

**Published:** 2022-04-14

**Authors:** Demóstenes Alves Diniz, Kalyne Kelly Negromonte Gonçalves, Caio César Gonçalves Silva, Emerllyn Shayane Martins de Araújo, Suzana Célia de Aguiar Soares Carneiro, Carlos Augusto Pereira do Lago, Belmiro Cavalcanti do Egito Vasconcelos

**Affiliations:** 1 Master’s Student in Oral and Maxillofacial Surgery (FOP/UPE). Specialist in Oral and Maxillofacial Surgery (HR/UPE). University of Pernambuco, Brazil; 2 PhD Student in Oral and Maxillofacial Surgery (FOP/UPE). Specialist in Oral and Maxillofacial Surgery (HR/UPE). University of Pernambuco, Brazil; 3 Resident in Oral and Maxillofacial Surgery (HUOC/FOP/UPE). University of Pernambuco, Brazil; 4 Staff of Service of Oral and Maxillofacial Surgery, Restauração Hospital (HR/FOP/UPE), Recife, Brazil; 5 DDS, MSc, PhD. Coordinator of Graduation Program (PhD and Master’s Degree) in Oral and Maxillofacial Surgery. University of Pernambuco. Head of Restauração Hospital. Pernambuco, Brazil

## Abstract

**Background:**

Liposuction is one of the most commonly performed cosmetic procedures worldwide. Complications associated with submental liposuction are rare. However, when they occur they are significant and can cause disFiguring consequences. The objective of this study was evaluated complications from submentual liposuction in literature and description of clinical experience of complication after submentual liposuction.

**Material and Methods:**

At first, a scoping review was carried out online search with no time restrictions for complications after submental liposuction was performed in the databases Medline / PubMed, Embase, and Web of Science. The variables analyzed were: age, sex, type of esthetic procedure, anesthesia, complications, time after Procedure, treatment, follow-up care, and sequelae. Then, a case of a patient with submental hematoma after an aesthetic procedure for submental liposuction was described.

**Results:**

Firstly, 539 articles were selected, after application of the inclusion criteria, 4 studies were included. Most cases were female (8:1), with a mean age of 55.77 years. Postoperative complications were found, such as submental depression, submental edema, hypertrophic scar formation, scar contracture, cervical necrotizing fasciitis, Cervico-facial dystonia and transient facial nerve paralysis. The follow-up period for cases ranged from 3 to 12 months. The clinical case presented there was no sequelae.

**Conclusions:**

Submental liposuction requires the surgeon's attention. Anatomical knowledge, correct clinical and surgical management, diagnosis, and immediate approach to adverse situations are points that must be respected in this type of esthetic procedure to avoid more serious complications.

** Key words:**Surgery plastic, lipectomy, postoperative complications, hematoma.

## Introduction

Liposuction, or suction lipectomy, is one of the most commonly performed cosmetic procedures worldwide. It is an excellent technique on face and anterior neck to remove fat deposits, usually at the subplatysmal level, improving the contour of these regions. When performed at the submental region it is through an insertion of an aspiration cannula through a small incision of 1 to 2 cm in the skin (1-3).

This approach can be performed at the hospital under general anesthesia or at an outpatient clinic, through association of sedation with local anesthesia or even under local anesthesia only, without systemic muscle relaxants or anxiolytic drugs. When performed under local anesthesia with conscious sedation, it has the advantage of the patient's cooperation in positioning his/her head, simplifying the procedure as there is no need for intubation and the trans and postoperative stability of blood pressure (4).

Complications associated with submental liposuction are rare. However, when they occur they are significant and can cause disFiguring consequences (5). It is one of the most feared complications is the formation of bruises, which can contribute to partial or total necrosis of the skin. The main risk factor for the appearance of bruises is correlated with the elevation of systolic blood pressure in the pre, trans or postoperative period due to anxiety and pain that increase adrenaline levels. In addition to esthetic damage, hematoma can cause airway obstruction and, consequently, the patient's death. Therefore, anatomical knowledge, correct surgical techniques, diagnosis and immediate management of complications are essential points for surgeons who deal with cosmetic procedures in the face (5,6).

Currently, there are not enough studies to define correct management for cases of complications after submental lipectomy, however, its forms of prevention have already become evident. In this context, the objective of this study is to carry out an scoping review of cases of complications after submental liposuction and to discuss details such as age, sex, clinical and surgical management, sequelae and methods of preventing these complications. Furthermore, the case of a patient with cervical hematoma after submental liposuction procedure is reported.

## Material and Methods

- Protocol and Registration

This scoping review was structured based on five steps methodology proposed by Arksey and O’Malley (2005) (7) and followed the ‘PRISMA Extension for Scoping Reviews (PRISMA-ScR)’ (8,9).

Step 1: Identifying the research question

Focal question: “What is the occurrence of complications associated with submental liposuction?”. OSF Registries protocol #osf.io/7sw5j.

Step 2: Identifying relevant studies

Literature searches were performed in the MEDLINE (via PubMed), Embase and Web of Science databases for articles published up to May, 2020, using MeSH, Emtree and DeCS/MeSH terms, and other free terms, combined by the Boolean operators "OR" and "AND": (“Liposuction” OR “Lipectomy” OR “Lipectomies” OR “Aspiration Lipectomy” OR “Aspiration Lipectomies” OR “Lipectomies, Aspiration” OR “Lipectomy, Aspiration” OR “Aspiration Lipolysis” OR “Lipolysis, Aspiration” OR “Suction Lipectomy” OR “Lipectomies, Suction” OR “Lipectomy, Suction” OR “Suction Lipectomies” OR “Lipolysis, Suction” OR “Suction Lipolysis” OR “Liposuction” OR “Liposuctions” OR “Lipoplasty” OR “Lipoplasties”) AND (“Submental” OR “Submental Fat”). The gray literature was accessed by consulting the OpenGrey, and the Biblioteca Digital Brasileira de Teses e Dissertações databases. Manual searches were also performed in the following specialized journals (step 1): The International Journal of Oral and Maxillofacial Surgery; Journal of Oral and Maxillofacial Surgery; British Journal of Oral and Maxillofacial Surgery; Journal of Cranio-Maxillo-Facial Surgery; The Journal of Dermatology; International Journal of Dermatology; British Journal of Dermatology; and Dermatology Surgery and Neurosurgery; and in reference lists of selected articles (step 2).

Step 3: Study selection

The controlled vocabulary (MeSH, Emtree and DeCS/MeSH terms) and free keywords in the search strategy were defined to identify descriptive or analytical studies on the occurrence of complications associated with submentual liposuction. Elements of the PECO question:

Participants (P) = patients submitted to submental liposuction

Exposition (E) = complications after submental liposuction

Control (C) = patients control group without complications, and no control group in descriptive studies

Outcomes (O) = demographic data, clinical and surgical management, sequelae and methods of preventing

Inclusion criteria: case reports, case series and retrospective or prospective studies involving complications on the submental region and / or neck after the cosmetic procedure of submental liposuction associated or not with other esthetic procedures, with no period of publication or language restrictions. Exclusion criteria: I - studies unrelated to the subject; and ii - unavailability of full paper copy. No time or language restrictions were applied.

The selection process was conducted in two phases: Phase 1, two researchers (D.A.D. and K.K.N.G.) independently examined the titles and abstracts of all identified references, applying the inclusion process (blind process); and Phase 2, the same two reviewers independently applied the exclusion criteria to the other studies based on reading the full text (blind process). Inter-reviewer reliability in the study selection process was determined by the Cohen κ test, assuming an accepTable threshold value of 0.80(10). The disagreement at any stage was resolved by discussion and mutual decision (consensus meeting) with a third reviewer (B.C.E.V.). The final decision/selection was always based on the full publication text.

Step 4: Charting the data

The full texts were evaluated and judged in the entire document. Authors were contacted when necessary to obtain details on study design and data clarification. The data from the eligibility forms were stored in Tables by independent double typing (D.A.D. and K.K.N.G.), and the validation process was conducted by a third reviewer (B.C.E.V.).

Step 5: Collating, summarizing and reporting the results

After mapping the information from the studies, we were able to present our narrative account of the findings in three ways: i) descriptive data reported in basic numerical analysis of the extent, nature and distribution of the studies included in the review; ii) individualized and combined information about the complications associated with submental liposuction; iii) response to treatment, impacts/complications arising from complications and its treatment, and follow-up; and iv) data related to the evidence level of the included studies.

The following data were extracted: author, year of publication, type of study, sex, age, type of esthetic procedure, type of anesthesia, postoperative complications, time elapsed after the procedure, treatment, monitoring and permanent sequelae (Table 1).

Descriptive data were stratified by the study design and the included studies were classified for level of evidence according to the Oxford Center for Evidence-Based Medicine (OCEBM, 2011).

- Patient issues

The patient gave written consent for publication of her case, in accordance with the Declaration of Helsinki.

## Results

- Study selection

**Figure 1:**
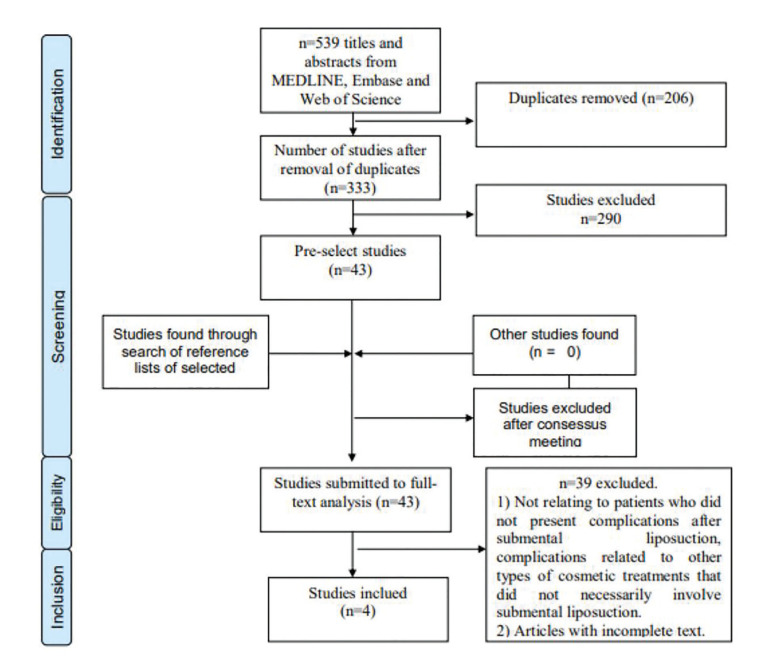
PRISMA flow diagram of the study selection process.

The scoping review resulted in 539 articles. After applying the inclusion and exclusion criteria and removing duplicates, 4 studies were selected for analysis and discussion (Fig. 1). The other articles were not considered due to the absence of case reports associated with complications after submental liposuction (Table 1).

- Sample

The sample consisted of nine patients. The female/male ratio was 8:1. The mean age was 55.77 years (range, 32 to 72 years). All cases were associated with postoperative complications of submental liposuction: two cases with submental depression (11), one case occurred swelling on the submental region (11), another case with hypertrophic scar formation occurred (11), plus two reports with a scar contracture (11). Three other studies reported cases of cervical necrotizing fasciitis (12-14), a case of cervico-facial dystonia and transient facial nerve paralysis (13). The follow-up period ranged from 3 to 12 months. Only one study did not report this information (11). As mentioned in Table 1, in all cases of submental depression that is necessary, the surgical approach was performed with a surgical technique of zetaplasty to correct deformity, in case 1 it was performed with double zetaplasty. The cases of bacterial infection were necessary in all cases of surgical approach for tissue debridement and use of intravenous broad-spectrum antibiotics.

- Ilustrative case

**Figure 2:**
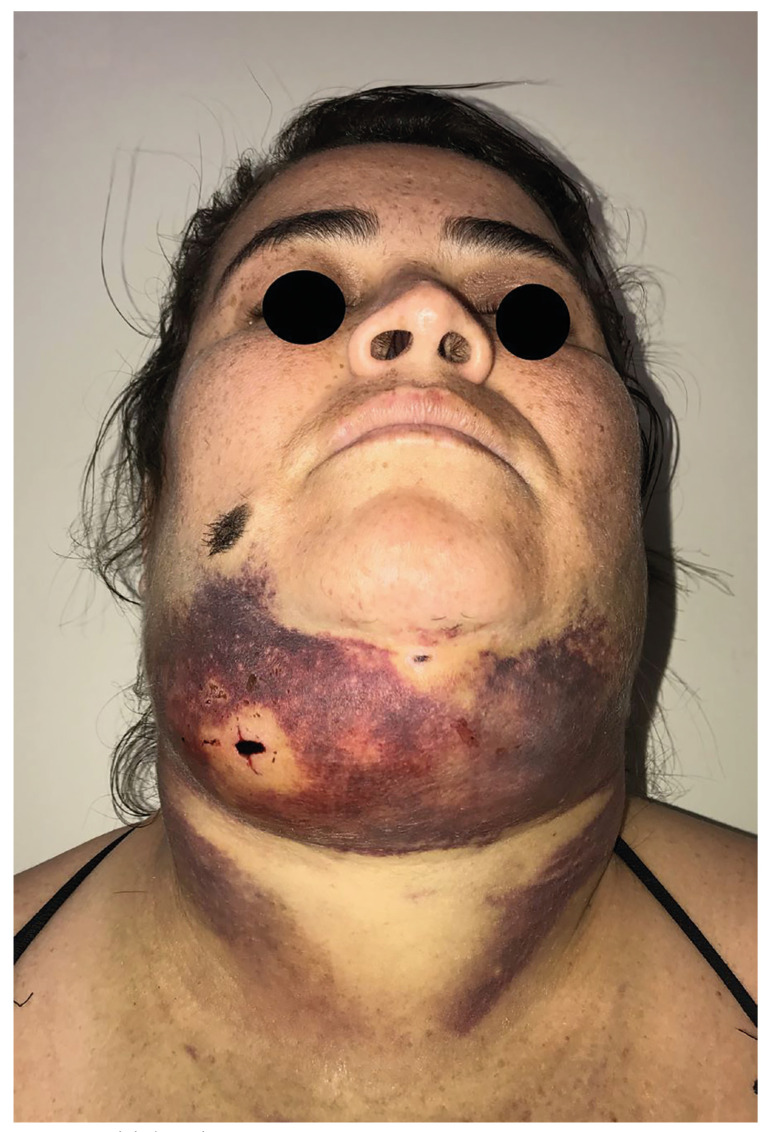
Initial patient care.

In November 2019, a 36-year-old woman attended the Oral and Maxillofacial Surgery service at the emergency room of Restauração Hospital, Recife - Pernambuco, with exuberant submental swelling after an aesthetic procedure (Fig. 2). During a clinical examination, a patient reported having undergone submental liposuction under local anesthesia (± 24 hours). The patient reported that she suffered from anxiety disorder, making continuous use of anxiolytics (Clonazepam 2mg); she denied chronic degenerative diseases, but reported family history of systemic arterial hypertension (SAH). During assessment of vital signs, tachycardia and hypertensive peak (180 x 120 mmHg) were found. On physical examination, she had significant hematoma on submental and bilateral submandibular region (cervical zone II) (15), presence of incision (± 5mm) on submental region, in addition to dysphagia and dysphonia.

**Table 1:**
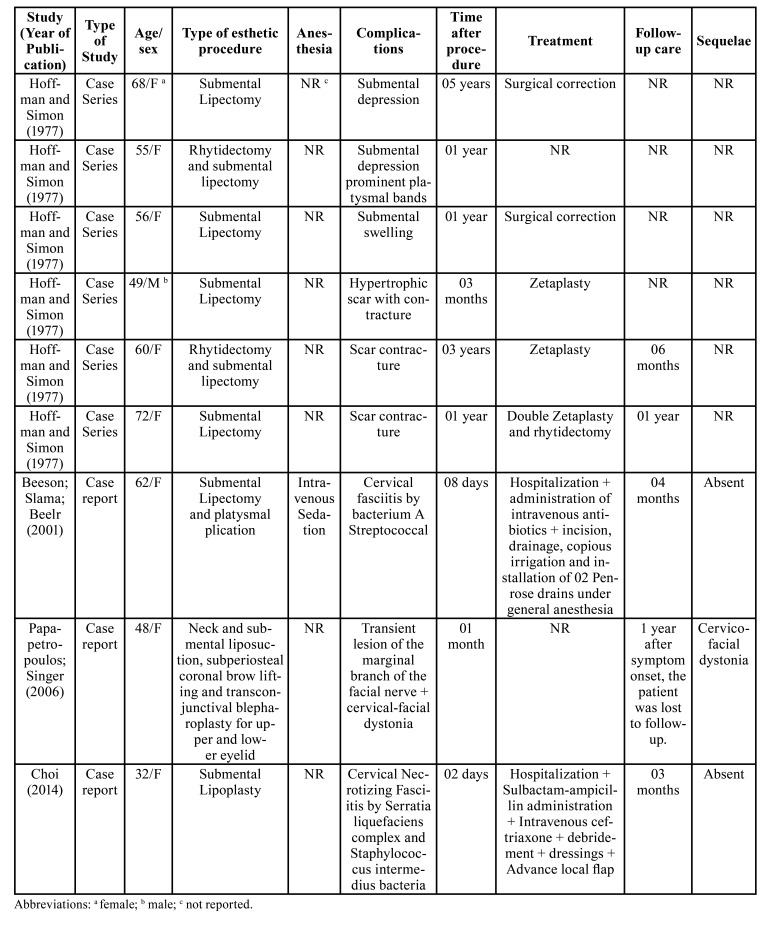
Literature case data that presented complications after submental liposuction.

**Figure 3:**
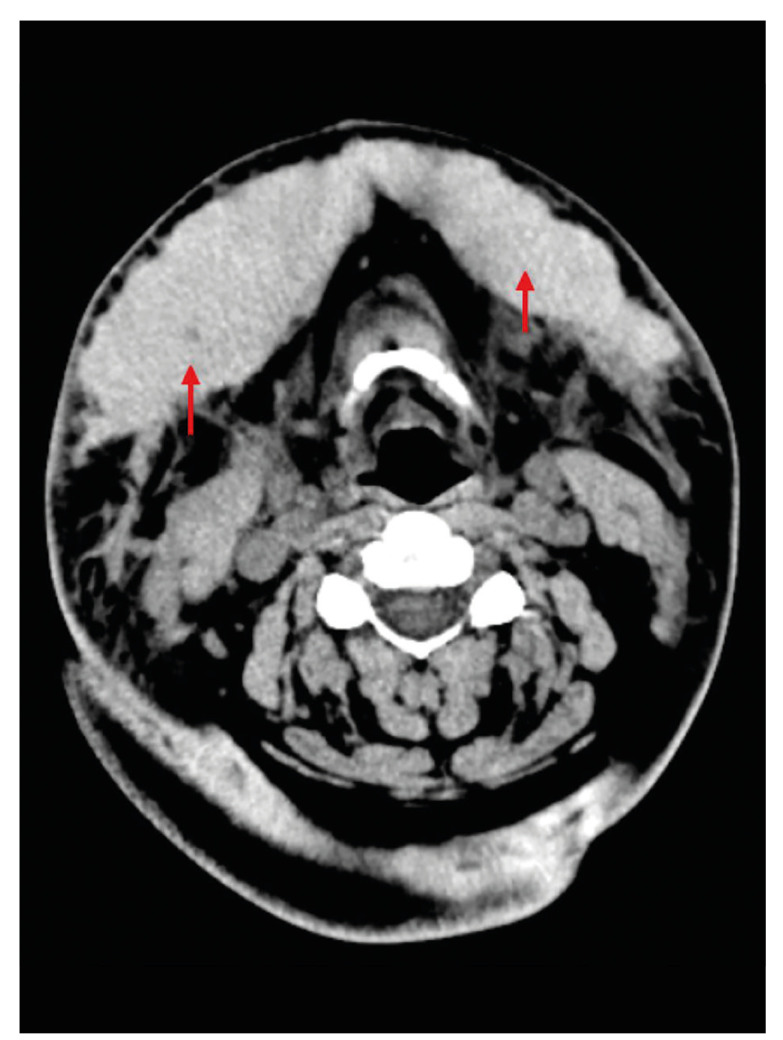
Computed Tomography (CT) showing large subcutaneous hematoma.

**Figure 4:**
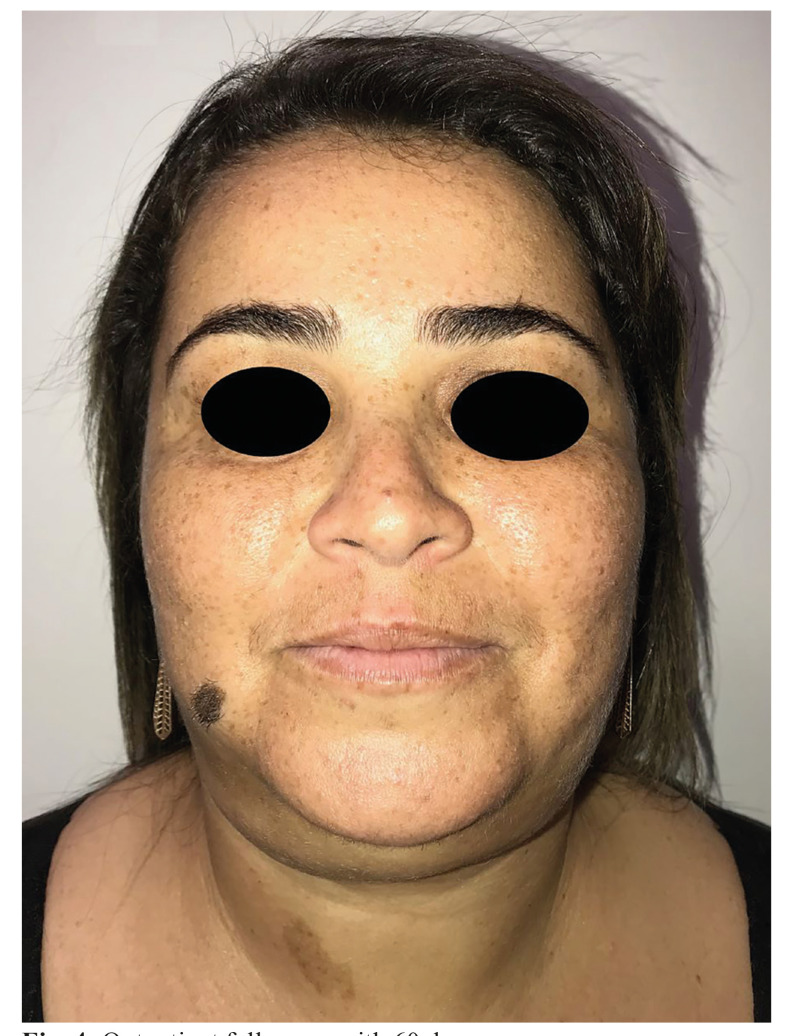
Outpatient follow-up with 60 days.

Ultrasonography of the soft tissues on submental region suggested the presence of a heterogeneous subcutaneous tissue collection, with distance between the collection center and cutaneous surface of approximately 2.0 cm, with predominantly hypoechoic content and some cystic intervening areas, presenting a maximum thickness of 2.5 cm on the right submandibular region and 1.6 cm on the left region, with a diagnostic hypothesis of hematic collection. Computed tomography of the face and neck showed the presence of large subcutaneous hematoma at level I Cervical, bilaterally bulkier on the left, superficial to cervical fascia, with measures of 11.1 x 3.6 x 4.9 cm, and an estimated volume of 102 ml (Fig. 3). Laboratory tests did not find the presence of coagulopathies or leukocytosis, however they suggested mild acute anemia (Hemoglobin of 10.6 g/dL, Hematocrit 31.6%), probably caused by volume loss after the surgical procedure. The patient underwent submentual hematoma drainage with a Penrose 2 drain under local anesthesia, as well as management of antibiotic therapy (Ceftriaxone 1g 12/12h - 7 days) and corticosteroid therapy (Dexamethasone 4mg 8/8h - 3 days) both intravenously. Thermotherapy with heat compresses and analgesia (Acetaminophen 500mg, Oral Route, 6/6h) were also managed. After 5 days the drain was removed and after the twentieth day the patient was in complete regression of the hematoma, in addition to clinical and laboratory improvement. The patient was discharged from the hospital and was followed up at an outpatient basis for 2 years without presenting functional and/or esthetic sequelae (Fig. 4).

## Discussion

This review showed that complications associated with submental liposuction are situations rarely reported in literature and that the main risk related to this procedure is formation of hematomas that can progress to tissue necrosis. As presented in this scoping review, female patients tend to be more affected (8: 1) and this fact can be justified by the more frequent search of women for esthetic procedures, aiming at improving their appearance and self-esteem (16). However, due to the small sample size of our study, this should be interpreted with caution.

The search for submental liposuction usually occurs in patients > 50 years old (1). In this scoping review’s studies, the participants had a mean age of 55.77 years. This fact suggests that esthetic complaints increase with senility, due to changes in skin elasticity, fat accumulation and loss of muscle tone. Therefore, because they are older, patients are more likely to have associated comorbidities, such as obesity, SAH and diabetes, and are therefore more subject to complications after surgical procedures (11,17).

In this sense, the esthetic procedures that seek rejuvenation of the face and neck strive to reverse signs of aging while optimizing the patient's natural anatomy, contributing to self-esteem and the notions of beauty standardization, causing increasingly younger patients to undergo these types of surgery. Common features of a pleasant lower face include a well-defined and properly balanced jaw and an acute cervicomental angle (6). The illustrative case in question corroborates this statement, since she was a 36 years old patient. Regarding obesity, the regular practice of physical exercise combined with a balanced diet only reduces the size of the adipose cells. The resistant fat is removed only by liposuction (18). Therefore, this procedure is not a treatment for obesity (19). It is recommended that patients undergoing submental liposuction are within 30% of their ideal Body Mass Index (BMI) (20). A study by Chow et. al. (2015) found that liposuction volumes greater than 100 ml per unit of BMI increase the risk of complications, including the formation of hematomas (21). Therefore, measuring this value in the preoperative evaluation is important, as well as suggesting to the patient the association of diet control, in addition to regular physical exercise, thus obtaining better results (19). To minimize complications, some factors must be taken into account when approaching the candidate for submental liposuction, such as age, presence of comorbidities, use of thrombolytic medications and psychological aspects. As the procedure is elective, the evaluation of motivation for choosing the treatment, recent life events and expected results with surgery must be scored (12,17). In our illustrative case, the patient had anxiety, so we assume that a procedure under sedation associated with local anesthesia would be more appropriate, minimizing the risk of trans operative hypertensive peak caused by anxiety and pain.

Furthermore, the personal and family history of possible blood dyscrasias should be investigated, such as a history of dysfunctional uterine bleeding, previous procedures that evolved with complications or hemorrhages after tooth extractions. These are worrying factors that deserve attention (22). In the illustrative case, the patient was unaware of the condition of SAH, however, she had a family history and reported that at the service where the surgical procedure was performed, blood pressure control was not performed at any time. Thus, the postoperative hypertensive peak may justify the formation of an expressive submental hematoma. From an anatomical and technical point of view, bleeding is certainly found during submental liposuction if muscle fibers are violated through the passages of the aspiration cannulas, occurring in cases where the muscle layer is disrespected, on the subplatysmal approach, thus offering damage to the main blood vessels in the neck. Hemostasis appears to depend on the surgeon's ability to keep the extractor tip inside the fat layer and extract the fat evenly and systematically (23).

Although the overall prevalence of complications is relatively low after submental liposuction, any complications that do occur can have devastating potential (16). The hematoma is the main complication associated with this esthetic procedure. The key to managing this hematoma is prevention and immediate treatment. An expansion of cervical hematoma carries risks, due to the potential for extrinsic compression of the airways. Untreated hematoma can promote skin necrosis, infections and unfavorable scarring, promoting contour deformities and aesthetic damage (17). Thus, as performed and guided by most cases in literature, the hematoma was immediately drained in an attempt to decompress the upper airways, improving the patient's complaints of dysphagia and dysphonia. The most common complication after any plastic surgery is hematoma, and the main risk factor is elevated systolic blood pressure (4). In order to justify the relationship between hypertensive peak and hematoma formation, it was observed in studies that a preoperative blood pressure above 150/100 mmHg was associated with 2.6 times higher incidence of hematoma (24). In this sense, the importance of monitoring patients' blood pressure levels is evident, and the patient's normotensive general state is supported for performing esthetic procedures.

In addition to hematoma, other complications may be associated with mechanical liposuction with cannulas on the submental area, such as depressions and deformities, generated by the excessive removal of subplatysmal fat, as the submental skin adheres to the mylohyoid muscle (25). This type of complication may have its onset later, as shown by this scoping review, one of the cases reported that a patient developed submental-cervical depression 5 years after the surgical procedure (11). Because of the aesthetic sequelae, we found cases that require a new surgical intervention, thus generating additional psychological stresses for patients (11). Moreover, laterally, lipectomy can cause damage to marginal nerve of the mandible and major auricular nerve and also damage the salivary glands, generating sialoadenitis (25). In this review, nerve injuries were not significant findings, being reported only in one study, in which transient facial nerve paralysis occurred after submental liposuction (13).

According to the results found in this scoping review, two patients presented scar contracture as a complication, being a kind of healing that restricts movement due to the junction of skin to underlying tissue, generating undesirable local sequelae that can be irreversible (11). This complication requires more elaborate procedures for correction, such as performing zetaplasty or double zetaplasty.

Characteristics of infectious manifestations after aesthetic procedures on the face usually appear a few weeks or months after surgery. The average period of bacterial incubation varies from 1 to 8 weeks, thus justifying the use of broad-spectrum antibiotics in the postoperative period, preventing formation of abscesses (26). In the present case, we opted for Ceftriaxone, a third-generation cephalosporin with an increased spectrum for gram negative bacilli, preventing future bacterial infections due to accumulation of hematic content. We also report that more serious infections can develop after submental liposuction, such as necrotizing cervical fasciitis (12-14), needing a more radical approach.

Finally, to minimize the risk of hematoma formation after submental liposuction, it is recommended that when the procedure is performed under general anesthesia, the anesthetist should be alerted to maintain blood pressure at its normal pressure level or within 10% baseline, not generating hypotension, as this fact can mask bleeding and promote a hypertensive peak rebound after surgical procedure, providing formation of bruises (23). Therefore, when performed under local anesthesia, it has the advantage of patient cooperation in relation to positioning the head, as well as greater control of normal blood pressure levels (4). In this scoping review, only one study reported intravenous sedation as the method used to perform the surgical technique (12).

In summary, the performance of submental liposuction requires attention from the surgeon. Anatomical knowledge, correct clinical and surgical management, diagnosis and immediate approach to adverse situations are points that must be respected in this type of esthetic procedure to avoid more serious complications.
